# Expression of MyoD, insulin like growth factor binding protein, thioredoxin and p27 in secondarily overacting inferior oblique muscles with superior oblique palsy

**DOI:** 10.1186/s12886-018-0793-3

**Published:** 2018-05-30

**Authors:** Yeon Woong Chung, Jun Sub Choi, Sun Young Shin

**Affiliations:** 1 Department of Ophthalmology, College of Medicine, St. Vincent’s Hospital, The Catholic University of Korea, Suwon, Republic of Korea; 2 Department of Ophthalmology & Visual Science, College of Medicine, Seoul St. Mary’s Hospital, The Catholic University of Korea, Banpo-daero 222, Seocho-gu, Seoul, 06591 Republic of Korea

**Keywords:** Oxidative stress, Inferior oblique muscle overaction, Superior oblique palsy, Thioredoxin, p27

## Abstract

**Backgound:**

To identify and compare specific protein levels between overacting inferior oblique (IO) muscles in superior oblique (SO) palsy patients and normal IO muscles.

**Methods:**

We obtained 20 IO muscle samples from SO palsy patients with IO overaction ≥ + 3 who underwent IO myectomies (IOOA group), and 20 IO samples from brain death donors whose IO had functioned normally, according to their ophthalmological chart review (control group). We used MyoD for identifying satellite cell activation, insulin-like growth factor binding protein 5 (IGFBP5) for IGF effects, thioredoxin for oxidative stress, and p27 for satellite cell activation or oxidative stress in both groups. Using immunohistochemistry and Western blot, we compared expression levels of the four proteins (MyoD, IGFBP5, thioredoxin, and p27).

**Results:**

Levels of thioredoxin and p27 were decreased significantly in the IOOA group. MyoD and IGFBP5 levels showed no significant difference between the groups.

**Conclusions:**

Based on these findings, the overacting IOs of patients with SO palsy had been under oxidative stress status versus normal IOs. Pathologically overacting extraocular muscles may have an increased risk of oxidative stress compared with normal extraocular muscles.

## Background

Inferior oblique (IO) muscle overaction may occur as a primary condition or develop secondarily to specific events such as superior oblique (SO) palsy. When myectomies were performed to weaken the IOs, some overacting IOs were very bulky but others were of apparently normal size. A previous study using magnetic resonance imaging (MRI) demonstrated that IO belly diameter increased on upward gaze nearly equally between patients with and without inferior oblique overaction (IOOA) [1]. Bagheri et al. [2] reported that there was no detectable correlation between IOOA and muscle position or circumference. However, little is known about the molecular and microscopic differences between overacting and normal IOs.

Extraocular muscles have different metabolic and structural components compared with other skeletal muscles, both molecularly and microscopically [3]. However, both extraocular muscles and other skeletal muscles generate free radicals with repetitive contraction, which can result in cellular oxidative damage in severe or prolonged state [4–6]. Myocytes generally contain a network of antioxidant defense mechanisms to reduce the risk of oxidative damage against increased reactive oxygen species [3]. Prolonged oxidative stress can result in reduced antioxidant capacity in extraocular muscles, and a previous report revealed that the medial rectus muscles (MRM) of patients with exotropia had a redox imbalance status compared with normal MRMs [6].

No previously reported study has compared extraocular muscles under continuously contracting conditions, as seen in IOOA due to SO palsy and normal extraocular muscles. Thus, this study was undertaken to investigate and identify any difference between pathologically overacting and normal IOs at the protein level using MyoD, IGFBP5, thioredoxin, and p27 as example proteins. The brief introductions of each protein are summarized as follows.

MyoD, which is located in a specialized niche between the myofiber sarcolemma and the the basal lamina, is an essential protein for satellite cell differentiation. Satellite cells represent 2–10% of total myonuclei [7, 8]. They are known to be. Satellite cells become activated and express the myogenic regulatory factors Myf5 and/or MyoD following injury or growth stimulus, proliferate and generate the myogenic progenitors which are needed for muscle regeneration [9–11] or become new muscle fibers. Thus, we can identify in which group the satellite cell is activated by measuring and comparing MyoD levels.

IGF is known to induce hypertrophy in cultured neonatal rat cardiomyocytes through a specific receptor [12]. IGFBP binds to IGF and regulates its half-life. Thus, IGFBP5 detected in extraocular muscles can help us to determine whether IGF has any influence on overacting or normal IOs.

Thioredoxin decreases, as an antioxidant, in an oxidative stress state [13, 14]. If IOOA is caused by oxidative stress, thioredoxin would be expected to decrease significantly in the IOOA group versus the control group.

P27 maintains or arrests the quiescent phase in the cell cycle, and decreases before cell division in stem cells like the satellite cells [15]. Recently, p27 was shown to play a role in an oxidative stress state [16, 17]. If IOOA is caused by hyperplasia or oxidative stress, a difference in p27 levels between the groups, in conjunction with a difference in MyoD or thioredoxin level, would be expected.

## Methods

The IOOA group consisted of 20 IOs obtained from patients with secondary IOOA ≥ + 3 from SO palsy. A portion of the IO (8.0 mm from the insertion) was resected during IO myectomy surgery. Approval to conduct this study was obtained from the Institutional Review Board of the Catholic Medical Center (#KC09TISI0365). Ethics, consent, permissions and approval were obtained with written documents by all participants prior to surgery. Approval to conduct and securing human tissue of this study was obtained from the institutional review board of the hospital and the study protocol adhered to the tenets of the Declaration of Helsinki. 20 normal IOs as the control group were obtained from age-matched donor eyes of individuals within 12 h after the brain death. Ethics, consent, permissions and approval were obtained with written documents by their guardians based on each donor’s intension of eyeball donation before brain death. Any subject who had history of eyelid or extraocular muscle surgery or disease, orbital diseases, and eyeball or periorbital trauma by their medical records was excluded.

All IOs were transferred to a portable tank filled with liquid nitrogen and immediately delivered from the operation room to the laboratory. Before preservation at − 80 °C, portions of secured IOs were separated and proteins for analysis were extracted within 1 h from securing IOs.

We selected four proteins for investigation; MyoD, insulin-like growth factor (IGF) binding protein 5 (IGFBP5), thioredoxin, and p27.

For immunohistochemistry, we first prepared formalin-fixed, paraffin-embedded tissue sections. Then the sections were deparaffinized with xylene and transferred to 100, 95, 70, and 50% alcohols in sequence, twice each for 3 min. For blocking of non-specific binding activity, each slide was incubated in 1% bovine serum albumin at room temperature for 10 min. After removing the blocking buffer, 100 μL of appropriately diluted primary antibodies (MyoD, IGFBP5 and p27; Santa Cruz Biotechnology, Santa Cruz, CA, USA, thioredoxin; Abcam, Cambridge, London, UK) were applied to the sections on the slides and the slides were incubated in a humidified chamber at 4 °C for overnight. After washing with PBS for 30 min, secondary antibody (rhodamine-conjugated, Santa Cruz Biotechnology, Santa Cruz, CA, USA) was applied for 1 h. After washing, the sections were stained with Hoechest 33,258 (Sigma-Aldrich, St. Louis, MO, USA). Then, a cover slip was mounted on the slide glass using mounting solution. The stained tissues were observed by fluorescence microscopy (Axio imager 2, Carl Zeiss GmBH, Zena, Germany).

Western blotting was performed to compare levels of the protein, MyoD, IGFBP, thioredoxin, and p27 protein levels between the two groups. Total protein was isolated from the IOs using RIPA buffer (25 mM Tris-HCl, pH 7.4, 1% Tween 20, 0.1% SDS, 0.5% sodium deoxycholate, 10% glycerol, 150 mM NaCl, 5 mM EDTA, 1 mM PMSF, 50 mM NaF, 1 mM Na_3_VO_4_, and 1 μg/mL of aprotinin, leupeptin, and pepstatin) and protein concentration was determined using a BCA protein assay kit (Pierce, Rockford, IL, USA). After separating 20 μg of protein using 10% sodium dodecyl sulfate polyacrylamide gel electrophoresis, it was transferred onto polyvinylidene difluoride membranes (Amersham Life Science, Cleveland, OH, USA) with approximately 5% skim milk in PBS-0.1% Tween 20 for blocking. Then, the primary antibodies (MyoD, IGFBP5 and p27; Santa Cruz Biotechnology, Santa Cruz, CA, USA, and thioredoxin; Abcam, Cambridge, London, UK) were reacted overnight in a 4 °C cold room, and the membrane was washed and reacted with horseradish peroxidase-conjugated goat anti-rabbit antibody (1:1000; Santa Cruz Biotechnology, Santa Cruz, CA, USA) and was developed with an enhanced chemiluminescence solution (Santa Cruz Biotechnology). Finally, bands on the X-ray film were quantitated by densitometry with β-actin as the protein loading control.

Using densitometry (Image Master VDS 2.0; Pharmacia Biotech Inc., San Francisco, CA, USA), we obtained the optical density of each protein band and divided them by the optical density of actin to obtain the relative optical density of each protein. The relative optical density of each protein was then compared between the IOOA and control groups.

Statistical analyses were performed using SPSS statistical software for Windows (SPSS, version 20.0, Chicago, IL, USA). Student’s t-test and the Mann-Whitney U test were used to evaluate differences between groups. A *P* value < 0.05 was considered to indicate statistical significance.

## Results

Twenty overacting IOs from 20 patients with SO palsy were obtained as the IOOA group (13 males, 7 females; mean age = 55.25 years). 20 IOs were obtained as the control group (6 males, 4 females; mean age = 55.25 years). There was no significant difference in gender or age between the two groups (Table 1).

**Table 1:** Demographic data of the IOOA and control group

	IOOA	Control	*P* value*
Number of patients (number of eyes)	20 (20)	10 (20)	
Age, years (SD)	52.35 (11.43)	55.25 (9.68)	0.438
Gender (M:F)	13:7	6:4	0.282
Deviation, prism diopters (SD)	26.2 (11.8)		

*Mann-Whitney test

**Fig. 1:**
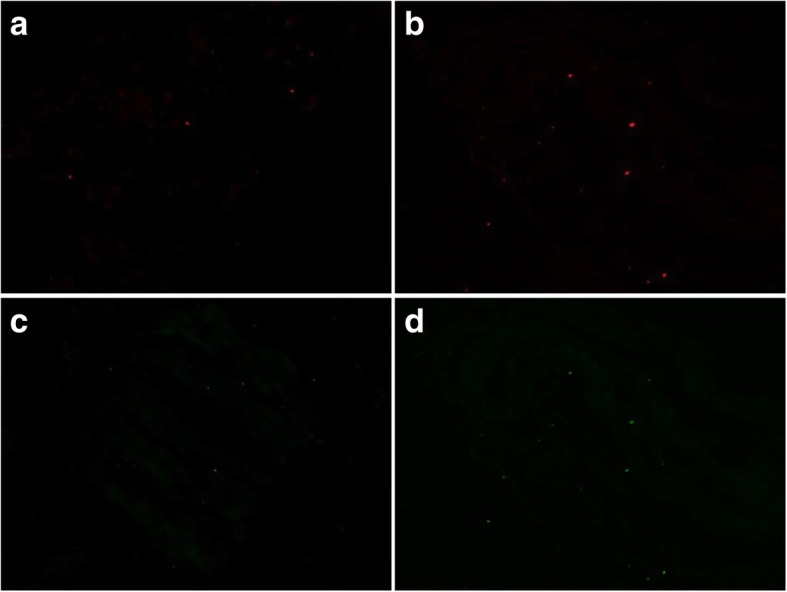
Immunohistochemistry results. MyoD (**a**) inferior oblique overaction (IOOA) group, (**b**) control group, insulin-like growth factor binding protein 5 (IGFBP5) (**c**) IOOA group, (**d**) control group

**Fig. 2:**
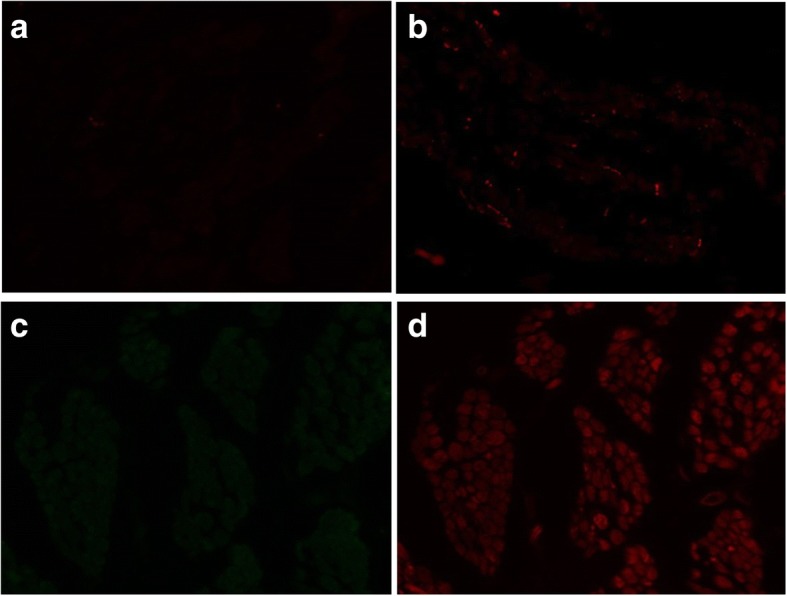
Immunohistochemistry results. Thioredoxin (**a**) IOOA group, (**b**) control group, p27 (**c**) IOOA group, (**d**) control group

In immunohistochemistry, few fluorescent dots representing MyoD and IGFBP5 were detected little in either groups (Fig. 1). However, many fluorescent dots for thioredoxin and p27 were detected in the control group but few dots were found out in the IOOA group (Fig. 2).

**Fig. 3:**
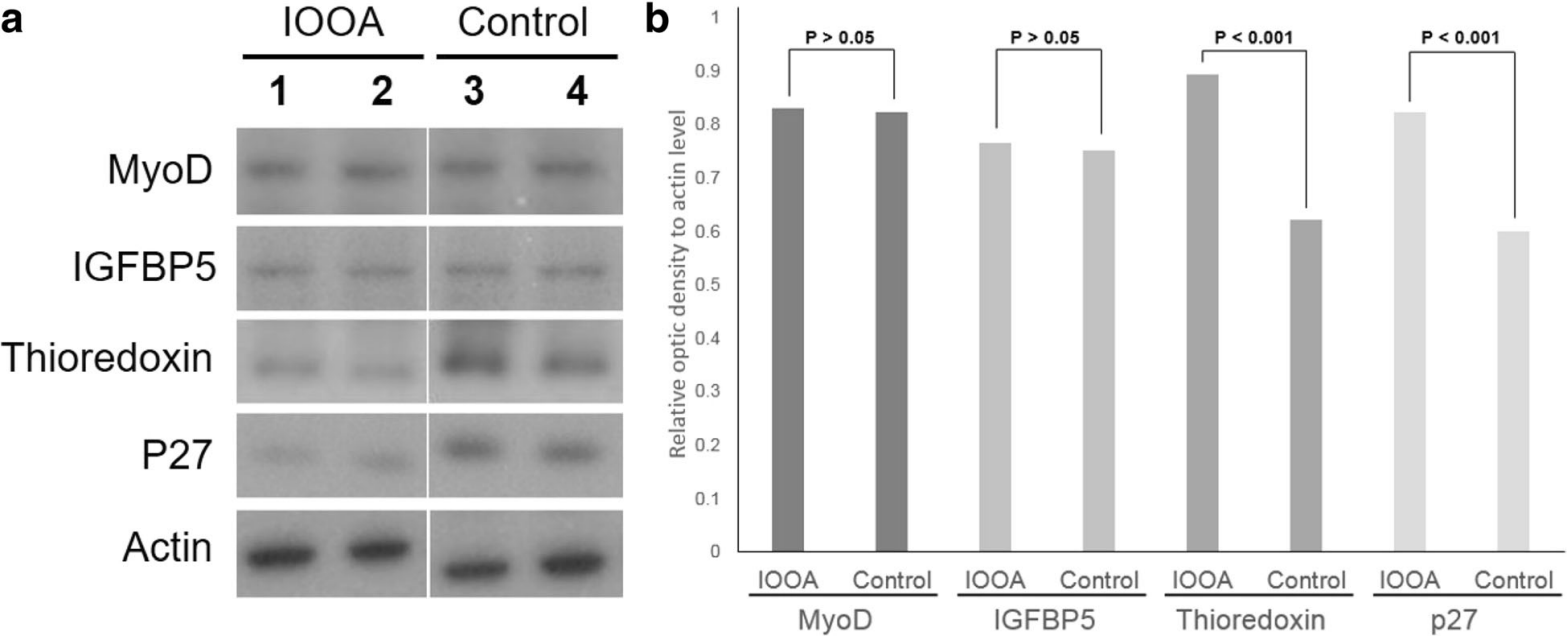
Western blot analyses of MyoD, IGFBP5, thioredoxin and p27. **a** Samples in the IOOA group are in lanes 1 to 2, and those in the control group are in lanes 3 to 4. **b** Levels of each protein in the IOOA group, compared with those in the control group. MyoD and IGFBP5 showed no statistical difference between the groups. However, thioredoxin and p27 were decreased significantly in the IOOA group versus the control group

On Western blotting and densitometry analysis, the levels of MyoD and IGFBP5 did not show a statistically significant difference (*P* > 0.05). However, the levels of thioredoxin and p27 were significantly higher in the control group than in the IOOA group in optical density (*P* < 0.001, respectively; Fig. 3).

## Discussion

Oxidative stress was defined as “a disruption of redox signaling and control” by Jones [18]. There are several methods for measuring oxidative stress in muscles and thioredoxin is a known antioxidant and an oxidative stress biomarker [14, 19]. To our knowledge, there is no previous report about thioredoxin levels in extraocular muscles. In the present study, thioredoxin was detected in the control group and was decreased significantly in the IOOA group. This indicates that thioredoxin is an antioxidant in extraocular muscles and there was a decrease in anti-oxidative capacity in the IOOA group.

A recent study showed another role for thioredoxin, in that its downregulation resulted in the induction of apoptosis [20] and its overexpression inhibited tumor necrosis factor-alpha (TNF-α) induced apoptosis, [21] indicating an anti-apoptotic role for thioredoxin. However, in our study, the actin levels between the IOOA and the control groups did not show a significant difference, indicating that there was no significant muscle fiber loss in the IOOA group. Thus, we consider the decreased level of thioredoxin in the IOOA group to be the result of the oxidative stress.

The decrease in p27 in the IOOA group, in conjunction with the decrease in thioredoxin, may also be evidence of oxidative stress. Sherr and Roberts [16] reported that downregulation of p27 in a particular condition suggested the presence of other mechanisms in regulating p27 levels, distinguishing it from cellular apoptosis. Lesley et al. [17] revealed that hydrogen peroxide and the antioxidant delphinidin seemed to regulate intracellular levels of p27 through regulating HIF-1 levels, which were, in turn, governed by its upstream regulators, involving the PI3K/Akt/mTOR signaling pathway. These findings support the hypothesis that oxidative stress and antioxidant regulate p27 by affecting PI3K-Akt signaling pathway.

Satellite cell activation, and the effects of intrinsic IGF on the IOs, were investigated through the levels of MyoD and IGFBP5 in the groups. However, they may have no or little effect in both the overacting and normal IOs although further study are needed to confirm this.

This study had some limitations. First, the IOs obtained from the IOOA group represented only a portion of the total IO length. Thus, the antioxidant capacity in the present study may not have represented the total capacity of IOs. To overcome this limitation, we included a similar length of the IOs from donor eyes. Second, the effects of satellite cell and IGF on the secondarily overacting IOs in the initial period remain unclear. IOs in the IOOA group were collected after prolonged overaction, caused by SO underaction, for at least several years. Third, the four proteins assessed in the present study do not represent the overall state of IOs.

## Conclusions

Secondarily overacting IOs in SO palsy are apparently in an oxidative stress state. In addition to our previous study that the MRMs of patients with exotropia had a redox imbalance status compared with normal MRMs, [6] extraocular muscles demanding continuous contraction may have pathological increased risk of oxidative stress compared with normal extraocular muscles.

## Abbreviations

IGF: Insulin-like growth factor
IGFBP5: Insulin-like growth factor binding protein 5
IO: Inferior oblique
IOOA: Inferior oblique overaction
MRM: Medial rectus muscle
SO: Superior oblique
